# MiR-106a-5p inhibits the cell migration and invasion of renal cell carcinoma through targeting PAK5

**DOI:** 10.1038/cddis.2017.561

**Published:** 2017-10-26

**Authors:** Yao-Jie Pan, Lu-Lu Wei, Xiao-Jin Wu, Fu-Chun Huo, Jie Mou, Dong-Sheng Pei

**Affiliations:** 1 Jiangsu Key Laboratory of Biological Cancer Therapy, Xuzhou Medical University, Xuzhou 221002, China; 2 Department of Oncology, The Affiliated Yancheng Hospital of Medicine School of Southeast University, Yancheng 224001, China; 3 Department of Pathology, Xuzhou Medical University, Xuzhou 221002, China; 4 Department of Radiation Oncology, The First People’s Hospital of Xuzhou, Xuzhou 221002, China; 5 School of Phamacy, Xuzhou Medical University, Xuzhou 221002, China

## Abstract

MicroRNA-106a-5p (MiR-106a-5p), a small non-coding RNA, has been reported to be downregulated in astrocytoma, osteosarcoma and colorectal cancer. However, the expression levels and biological function in renal cell carcinoma (RCC) have not been studied yet. In this study, we found that the miR-106a-5p was significantly downregulated in RCC tissues and cell lines, and that overexpression of miR-106a-5p led to decreased cell metastasis ability in a xenograft model. Inhibition of miR-106a-5p in RCC cell lines altered the cell migration, invasion and wound healing abilities. Mechanistic studies demonstrated that miR-106a-5p directly bound to the 3′-UTR of the PAK5 mRNA and mediated a decrease in the protein expression of PAK5. We further proved that PAK5 protein levels were negatively correlated with the miR-106a-5p expression in both patient samples and xenograft model. In epigenetics, methylation specific PCR experiments indicated that the upstream gene promoter of miR-106a-5p was hypermethylated in RCC, which might be responsible for its downregulation. Our findings suggested that miR-106a-5p might be a potential gene therapy target for the treatment of RCC metastasis.

Renal cell carcinoma (RCC) is the most lethal type of genitourinary cancer, accounting for 90% of renal malignancies, with an annual increase in incidence by 2–3%.^1, 2^ Despite the mainstay regimen of surgical resection, the overall effect remains unsatisfactory with a less than 50% 5-year post-operative survival rate, given the poor responsiveness of RCC to radiotherapy and chemotherapy.^3, 4^ The rate of distant metastases has developed up to circa one-tenth of RCC patients, which seriously reduces the clinical treatment effects and prognosis of RCC patients.^5^ Notwithstanding cytokine interleukin-2 (IL-2) and interferon (IFN) emerged as the stand care for metastatic RCC patients in recent years, their limited efficacy and substantial toxicity rarely benefit patients with an extensive tumor burden and adverse prognostic factors,^4, 6^ which craves the exploration of the mechanism underlying the tumor pathogenesis and potent approaches to RCC metastasis.

MiRNAs, which are small non-coding RNAs with 20–23 nucleotides, regulate the gene expression by binding to target mRNA in the location of 3′-untranslated regions (3′-UTR), leading to translational inhibition or mRNA degration.^7, 8^ MiRNAs are critical controllers of biological activities, such as embryogenesis and development, cell cycle, differentiation, apoptosis and oncogenesis.^9, 10^ Moreover, miRNAs are validated to be intricately involved in the regulation of tumor metastasis and epithelial-mesenchymal transition.^11, 12, 13, 14, 15, 16^ MicroRNA-106a-5p (MiR-106a-5p), a member of the miR-17 family, has been reported to be aberrantly regulated in a diversity of tumors. It is considerably downregulated and proved to exert tumor suppressor effects in astrocytoma, osteosarcoma and colorectal cancer.^17, 18, 19, 20^ However, the biological function of miR-106a-5p in RCC remains unclear. Therefore, identification of the effects of miR-106a-5p and its targets in RCC may lead to new perspectives for gene therapy clinical trials.

In our study, we examined the expression levels of miR-106a-5p in different RCC cell lines and tissues. Our data revealed that miR-106a-5p expression levels were significantly downregulated in RCC tissues compared with normal adjacent tissues, and the hypermethylated levels of miR-106a-5p gene promoter region might be responsible for the downregulation in RCC cell lines. Inhibition of miR-106a-5p was associated with increased cell migration, invasion and wound healing. Moreover, miR-106a-5p was able to function as an ‘anti-oncomiR’ by directly targeting the oncogene PAK5 and there existed an inverse correlation between miR-106a-5p and PAK5 expression levels. In addition, PAK5 overexpression partially abolished the effects of miR-106a-5p in the cell migration and invasion of RCC cells.

## Results

### MiR-106a-5p is downregulated in renal cell carcinoma

**Figure 1:**
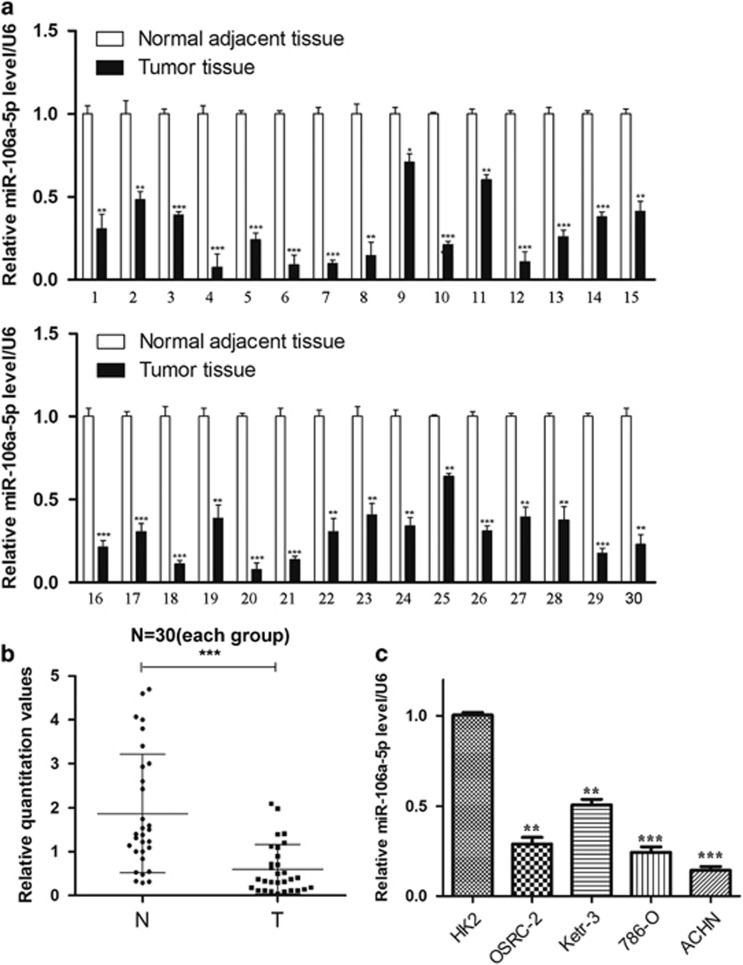
MiR-106a-5p is downregulated in renal cell carcinoma. qRT-PCR analysis of the miR-106a-5p levels in tissues and cell lines.(**a** and **b**) Relative miR-106a-5p expression in 30 paired RCC tissues and normal adjacent tissues. U6 was used to normalize (N represents the normal adjacent tissue, T represents the tumor tissue). The statistical significance was evaluated by paired-samples *T* test (*P*<0.001). (**c)** Relative miR-106a-5p expression in four RCC cell lines and normal renal tubular epithelial cell line HK2. U6 was used as an internal control. All the experimental results have been carried out for three times. The data represents the means±S.D. (*P<0.05, **P<0.01, ***P<0.001)

Previous reports indicated that miR-106a-5p was downregulated in astrocytoma, osteosarcoma and colorectal cancer. Here we intended to explore the expression levels of miR-106a-5p in renal cell carcinoma (RCC). The levels of miR-106a-5p were detected in 30 cases of RCC tissue samples and the normal adjacent tissues by qRT-PCR (Supplementary Table 1). Our results showed that miR-106a-5p levels were downregulated in tumor samples (100%) *versus* the normal adjacent tissues (Figures 1a and b). We next examined the miR-106a-5p expression in four human RCC cell lines (OSRC-2, Ketr-3, 786-O, ACHN) and normal renal tubular epithelial HK2 cells by qRT-PCR. The results indicated that the expression levels of miR-106a-5p were lower significantly than in HK2 cells wherein 786-O and ACHN possessed much lower miR-106a-5p levels (Figure 1c). Therefore, we used 786-O and ACHN cells as models to investigate the effect of miR-106a-5p on cell migration and invasion.

### MiR-106a-5p inhibits the migration and invasion of RCC cell lines

**Figure 2:**
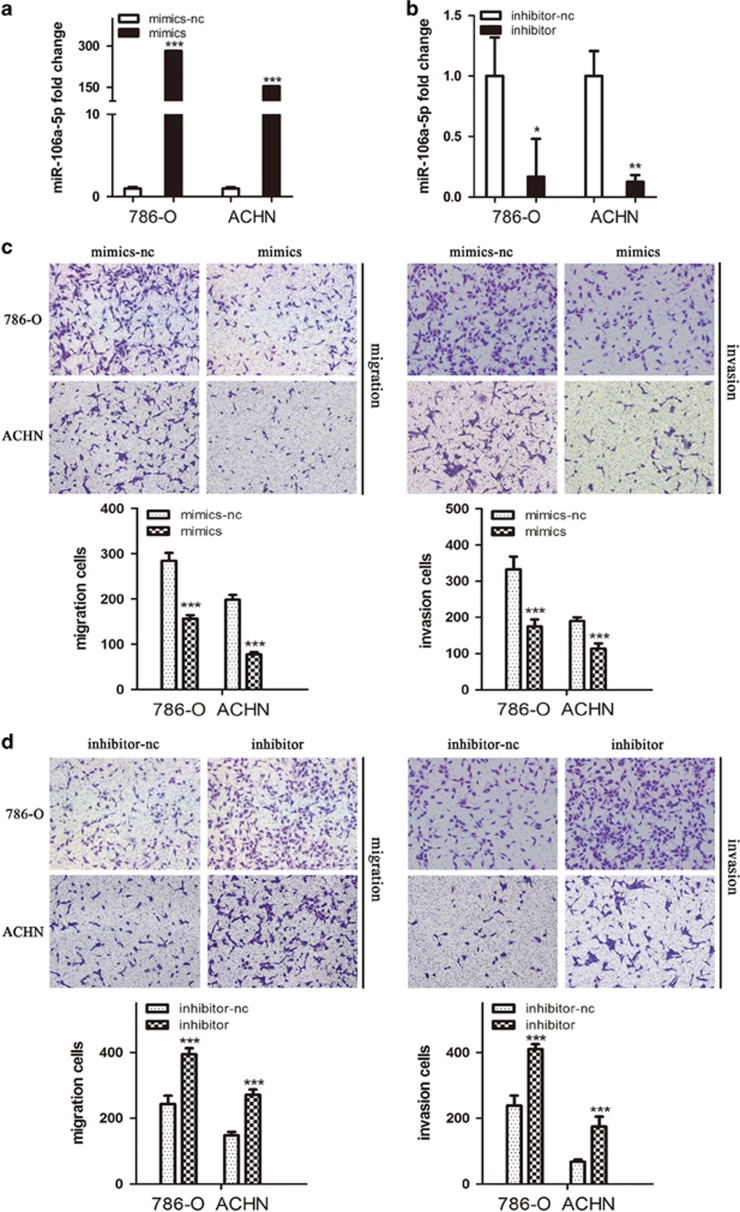

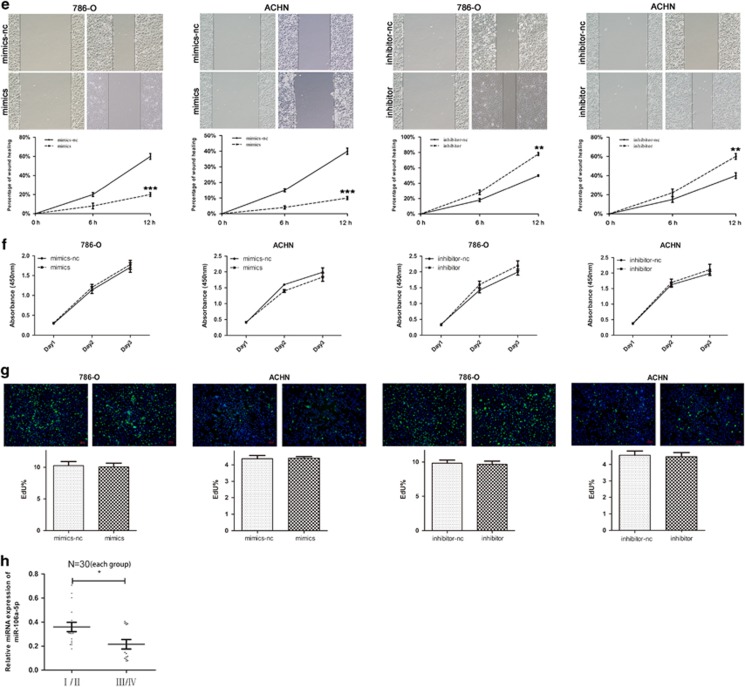
MiR-106a-5p inhibits the cell migration and invasion as well as wound healing. (**a** and **b**) Conformation of the levels of miR-106a-5p in transient transfected 786-O and ACHN cell lines by qRT-PCR; cells transfected with mimics-nc were used as the negative control. (**c** and **d**) The cell migration and invasion of 786-O and ACHN cells after transfection with mimics or inhibitor and their negative control (-nc) detected by transwell assays. (**e)** Representative images of the cell wound healing distance of shift after transfection with mimics or inhibitor and their -nc control. (**f**) The cell proliferation was detected by CCK-8 assays after transfection of mimics or inhibitor with respective -nc control, at 24, 48, 72 h. (**g**) Representative images of EdU staining after transfection of mimics or inhibitor with respective control. The EdU% represents the proportion of EdU-positive cells (Green). **(h)** Relative miR-106a-5p expression in 30 paired RCC tissues and normal adjacent tissues as the same set from Figure 1 with early TNM stages (I/II) and advanced stages (III/IV). All experiments were performed in thrice. The data represents the means±S.D. (*P<0.05, **P<0.01, ***P<0.001)

To assess the role of miR-106a-5p in the cell migration and invasion of RCC, transient transfection of miR-106a-5p-expressing cell lines were prepared, wherein the miR-106a-5p mimics (mimics) and miR-106a-5p inhibitor (inhibitor) were used as mediators for gain and loss of function studies, respectively. The levels of miR-106a-5p in the transient transfection cell lines were detected by qRT-PCR, and our results demonstrated the effectivenesses of transfections (Figures 2a and b). Cell migration and invasion were measured using transwell assays, and we observed that mimics-transfected 786-O and ACHN cells harboured weaker abilities to penetrate through the inserts without or with matrigel (Figure 2c). On the contrary, miR-106a-5p inhibition transfected with the inhibitor increased the number of cells passing through the inserts (Figure 2d). The results elucidated that miR-106a-5p could inhibit cell migration and invasion in 786-O and ACHN cells.

To examine the effect of miR-106a-5p upon cell wound healing, the cells that were grown to confluence in 6-well plates were wounded by scratching the cell monolayer with a disposable 200 *μ*l pipette tip. The shift of wound healing were monitored using a digital camera, and we found that mimics-transfected 786-O and ACHN cells exhibited a shorter distance of shift whereas inhibitor-transfected groups showed longer shift when compared with respective negative control group (Figure 2e). Our results indicated that miR-106a-5p could inhibit RCC cells wound healing ability.

Next, we discussed the function of miR-106a-5p on cell proliferation using CCK-8 assays and EdU staining. However, no significant changes of absorbance (Figure 2f) or green fluorescence (Figure 2g) were observed after mimics- or inhibitor-transfection, revealing that miR-106a-5p could not affect the proliferation of RCC cell lines.

Given that miR-106a-5p could inhibit the cell migration and invasion of RCC, we carried out statistical analysis to figure out whether the the stages of TNM (I/II/III/IV) were related with the differences of miR-106a-5p levels in RCC tumor tissues from the same set of specimens in Figure 1. Our results showed that the relative expressions of miR-106a-5p in tumor tissues were much lower in stage III/IV than those in stage I/II (Figure 2h, *P<0.05), indicating that miR-106a-5p was related with tumor progression of RCC.

### MiR-106a-5p directly targets the 3′-UTR of PAK5

**Figure 3:**
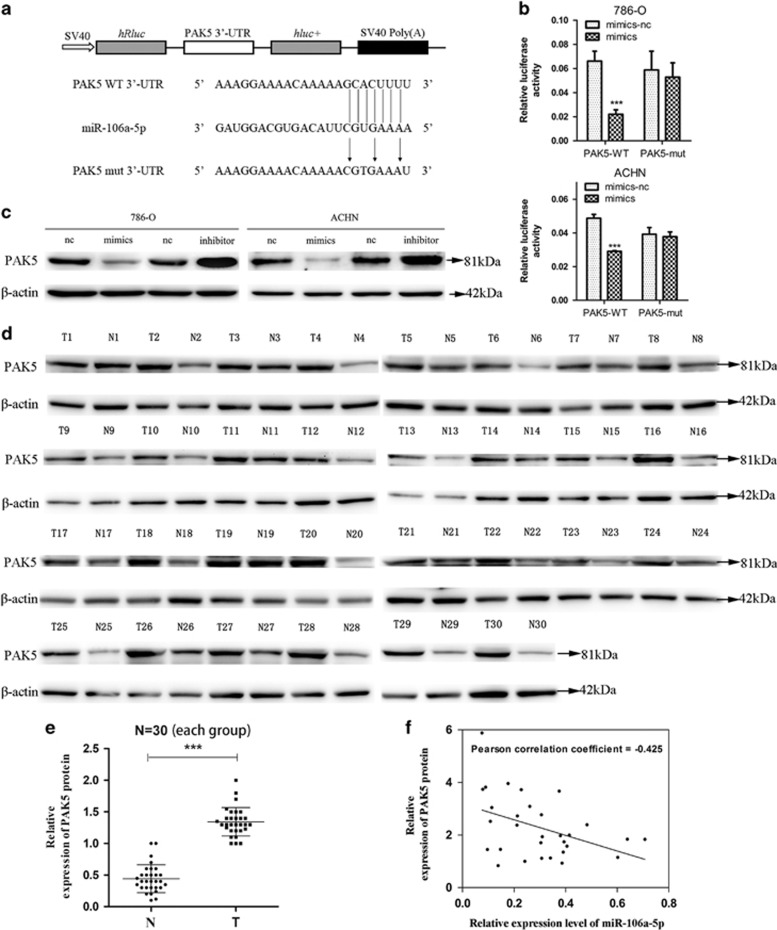
MiR-106a-5p downregulates the expression of PAK5. **(a)** Schematic description of wild type (WT) and mutated 3′-UTR of the PAK5 mRNA. The WT and mutated 3′-UTR sequences (264 bp) were cloned into the psiCHECK2 vector. (**b**) Dual luciferase reporter assay was used to detect the reporter activity. 786-O and ACHN cells in 24 well plates were co-transfected with 200 ng mimics-nc/mimics and psiCHECK2-PAK5 WT 3′-UTR/psiCHECK2-PAK5 mut 3′-UTR (100ng) for 48 h and then subjected to luciferase assays according to the Material and Methods (***P<0.001). (**c)** The PAK5 protein levels were measured by western blot analysis in 786-O and ACHN cells transfected with mimics-nc/mimics and inhibitor-nc/inhibitor. (**d**) Western blot of PAK5 protein levels in 30 paired RCC tissues, *β*-actin served as the loading control. (**e**) The relative expression levels of PAK5 protein, the T-axis values represent the grayscale value of PAK5/*β*-actin from N and T groups (***P<0.001). (**f**) Inverse correlation between miR-106a-5p and PAK5 in RCC tissues. MiR-106a-5p levels were normalized to the U6 levels, and the PAK5 levels were normalized to the *β*-actin levels. Statistical analysis was performed using Pearson’s Correlation Coefficient analysis (*n*=30, r=-0.425, *P*=0.0191). The data represents the means±S.D.

Previous studies reported that PAK5 was an important regulator of cell migration, invasion and tumorigenicity.^21, 22, 23, 24^ To explore the molecular mechanism responsible for the function of miR-106a-5p in RCC and whether PAK5 was directly targeted by miR-106a-5p, TargetScan (http://www.targetscan.org/) was employed to verify its possibility and the results presented a 97 context ++ score percentile, which was further supported by miRDB (http://www.mirdb.org/) and miRTarBase (http://mirtarbase.mbc.nctu.edu.tw/). The PAK5 wild-type 3′-UTR (PAK5 WT 3′-UTR) was cloned into psiCHECK2 vector (psiCHECK2-PAK5 WT 3′-UTR), following the open reading frame of upstream luciferase. In order to ulteriorly substantiate target specificity, site-directed mutagenesis for PAK5 WT 3′-UTR was conducted (PAK5 mut 3′-UTR, Figure 3a). Therein, we replaced guanine (G) with cytosine (C), uracil (U) with adenine (A), A with thymine (T) and C with G. The relative luciferase activities of the reporter gene in 786-O and ACHN cells co-transfected with psiCHECK2-PAK5 WT 3′-UTR and miR-106a-5p mimics were notably decreased by at least 30% compared with their control (co-transfected with psiCHECK2-PAK5 WT 3′-UTR and miR-106a-5p mimics-nc). Contrariwise, co-transfection of miR-106a-5p mimics-nc or mimics with psiCHECK2-PAK5 mut 3′-UTR resulted in insignificant alterations in luciferase activities, validating the miRNA/target 3′-UTR specificity (Figure 3b).

To further detect the protein levels of PAK5, we performed western blot analysis in transient transfected 786-O and ACHN cells. The results indicated that PAK5 protein expression levels were impaired significantly under conditions of miR-106a-5p overexpression. Conversely, inhibition of miR-106a-5p resulted in marked up-regulation of the PAK5 expression (Figure 3c). To discuss the correlation between miR-106a-5p expression and PAK5 protein levels, we analyzed the relative expression levels of PAK5 protein in the same set of specimens in Figure 1a by western blot analysis (Figure 3d). The results showed that the tumor tissue group (T) possessed higher expression levels of PAK5 protein than normal adjacent tissue group (N) (Figure 3e), and the miR-106a-5p levels were inversely correlated with PAK5 expression levels (*n*=30, r=−0.425, *P*=0.0191, Pearson’s correlation, Figure 3f). These data suggested that PAK5 was a direct target of miR-106a-5p in RCC.

### Inhibition of cell migration and invasion by miR-106a-5p via PAK5

**Figure 4:**
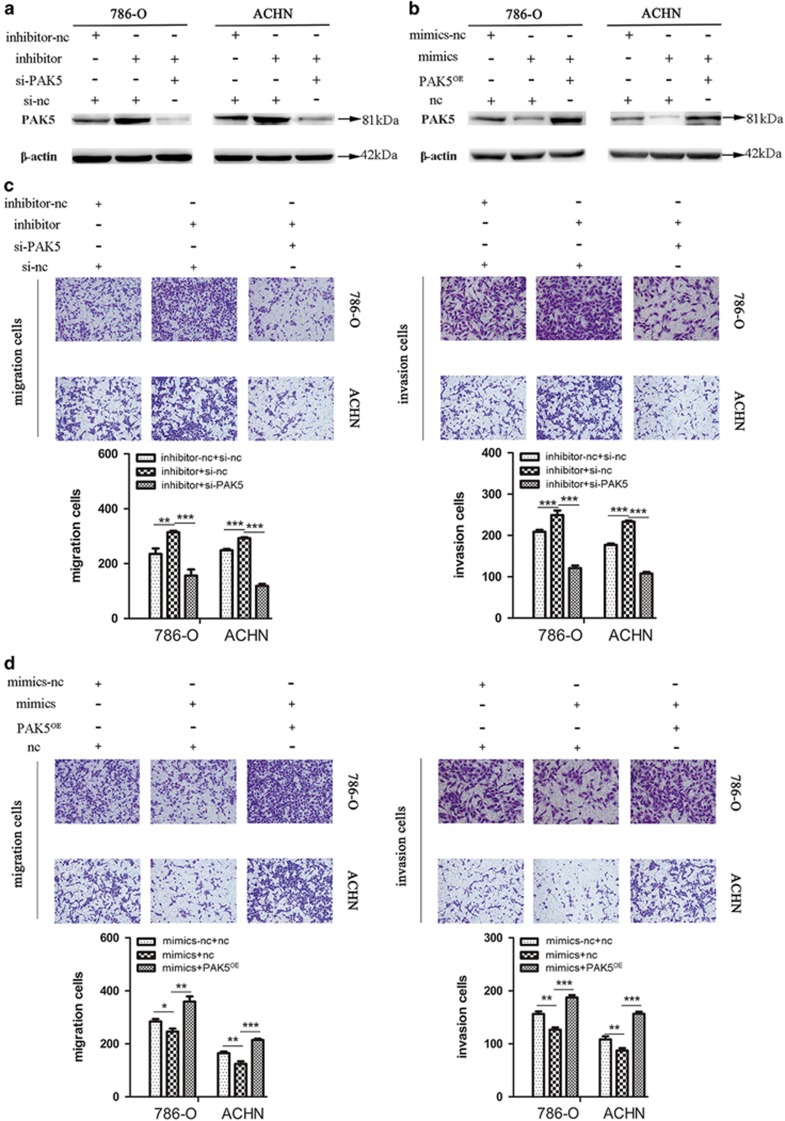
MiR-106a-5p regulates the cell migration and invasion by PAK5. **(a** and **b)** Conformation of the expression of PAK5 protein in three groups of co-transfection as labeled, *β*-actin was used as an internal control. (**c)** The cell migration and invasion detected by transwell assays after three groups of co-transfection (inhibitor-nc+si-nc, inhibitor+si-nc, inhibitor+si-PAK5) in 786-O and ACHN cells. **(d)** The cell migration and invasion detected by transwell assays after three groups of co-transfection (mimics-nc+nc, mimics+nc, mimics+PAK5^OE^) in 786-O and ACHN cells. All experiments were performed in triplicate. The data represent means±S.D. (*P<0.05, **P<0.01, ***P<0.001)

Our aforementioned results indicated that miR-106a-5p inhibited the cell migration and invasion and PAK5 was a direct target, next we discussed whether miR-106a-5p affected the cell migration and invasion of RCC cells via PAK5. Small interfering RNA (si-PAK5) was transfected to knockdown PAK5 for further experiments and the results from western blot showed that si-PAK5 worked efficiently in our system in Supplementary Figure 1. We conducted three groups of co-transfection and western blot analysis verified the effectiveness of co-transfection (Figures 4a and b). Transwell assays were applied to detect the cell migration and invasion and the results showed that following transfection with inhibitor, the numbers of migratory and invasive cells were significantly increased in contrast to inhibitor-nc group. Moreover, the trend of increase could be somewhat abolished by co-transfection with si-PAK5 rather than with negative control (si-nc; Figure 4c). Inversely, subsequent to transfection with mimics, the populations of migratory and invasive cells were decreased *versus* mimics-nc group, whereas the diminishing trend could be rescued by co-transfection with pcDNA3.1-PAK5 plasmids (PAK5^OE^) rather than with pcDNA3.1 empty vector plasmids (nc; Figure 4d). Our results demonstrated that miR-106a-5p could inhibit the cell migration and invasion of RCC cells via PAK5.

### Metastasis suppression of RCC cells by miR-106a-5p in xenograft model

**Figure 5:**
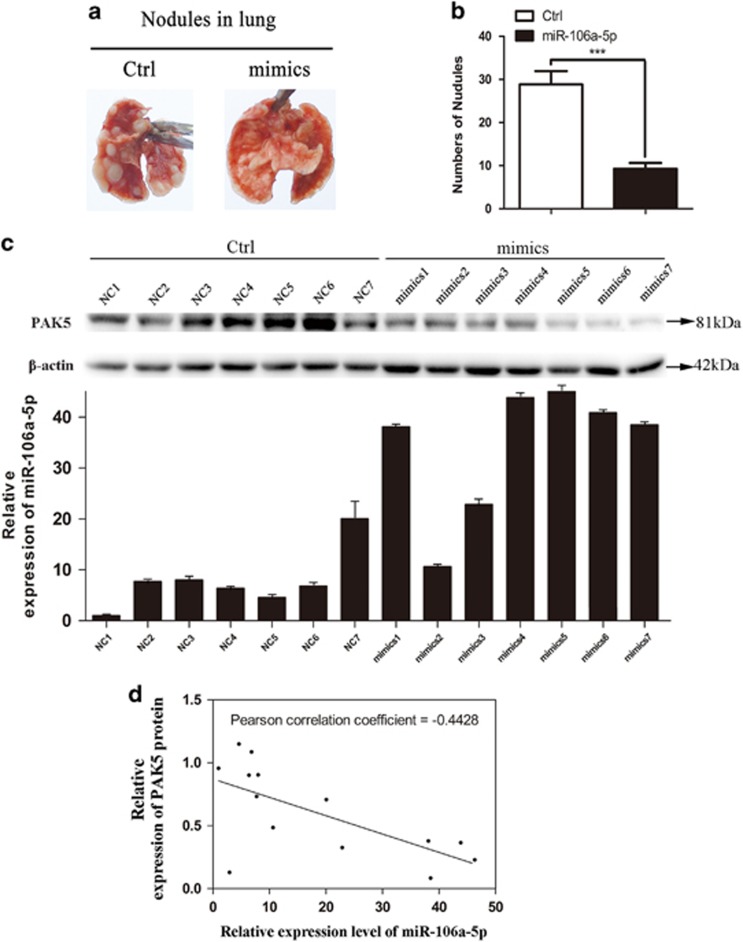
MiR-106a-5p suppresses RCC metastasis *in vivo*. **(a)** Representative images of lungs with metastatic nodules 2 months after respective injection of Ctrl, mimics groups cells. (**b)** The number of lung metastatic nodules was counted under a dissection microscope. A statistically dramatic reduction in the number of the lung metastases was observed in mimics group, compared with the Ctrl group. Data are displayed with means±S.D. for 7 mice in each group (***P<0.001). (**c)** Top panel, western blot analysis of PAK5 in 14 nude mice. Bottom panel, qRT-PCR analysis of miR-106a-5p expression in 14 nude mice. (**d)** Inverse correlation between miR-106a-5p and PAK5 in the lung nodules from 14 nude mice. MiR-106a-5p levels were normalized to the U6 levels, and the PAK5 levels were normalized to the *β*-actin levels. Statistical analysis was proceeded using Pearson’s Correlation Coefficient analysis (*n*=14, *r*=−0.4428, *P*=0.0094). The data represents the means±S.D.

To further validate the functional role of miR-106a-5p in RCC metastasis *in vivo*, lentiviruses packed with miR-106a-5p mimics-nc or miR-106a-5p mimics expression vectors were transfected into 786-O cell lines, namely Ctrl-786-O and miR-106a-5p-786-O cells. Fourteen nude mice were divided in two groups (*n*=7 for each) followed with injections of Ctrl-786-O (Ctrl group) or miR-106a-5p-786-O (mimics group) cells via tail vein, respectively. 2 months afterwards, the xenograft models of stable-transfected 786-O cell line were constructed. All mice were killed followed with the lungs being isolated for photography. Our results showed that metastatic tumor nodules in lungs of mimics group were significantly diminished in number *versus* Ctrl group (Figures 5a and b).

To detect the miR-106a-5p expression and PAK5 protein levels *in vivo*, the isolated lungs were then stored in RNA sample conservation medium (Vicmed, Xuzhou, China) at 4 °C, with the total RNA and protein extracted using Trizol Reagent within 24 h, followed by the qRT-PCR and western blot analysis. The results indicated that the miR-106a-5p expression levels were upregulated in mimics group *versus* Ctrl group (Figure 5c, bottom panel), and the protein levels of PAK5 were downregulated in mimics group *versus* Ctrl group (Figure 5c, top panel). Furthermore, a negative correlation between the expressions of PAK5 and miR-106-5p was revealed by statistical analysis (*n*=14, *r*=−0.4428, *P*=0.0094, Pearson’s correlation, Figure 5d). These suggested that miR-106a-5p could inhibit the lung metastasis of RCC *in vivo,* and the miR-106a-5p level was inversely correlated with PAK5 expression.

### The correlation of downregulated miR-106a-5p with the hypermethylated miR-106a-5p gene promoter region in RCC

**Figure 6:**
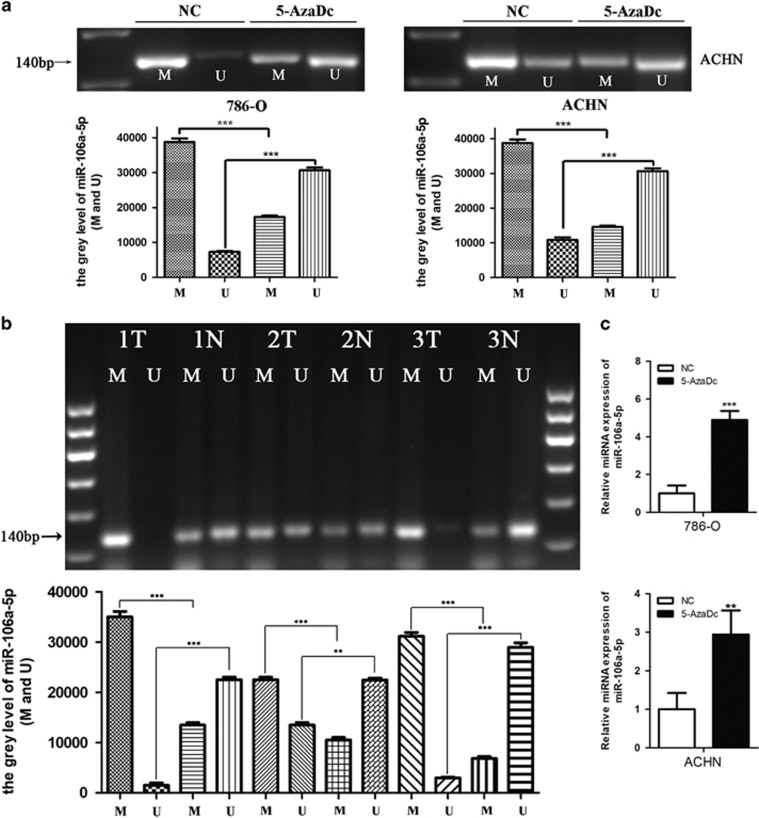
The downregulation of miR-106a-5p in RCC might be due to the hypermethylation of the miR-106a-5p gene promoter region in RCC tissues and cell lines. (**a)** The methylated and unmethylated miR-106a-5p levels in 786-O and ACHN cells were measured by MS-PCR analysis. M represents the methylated and U represents the unmethylated miR-106a-5p. (**b)** The methylated and unmethylated miR-106a-5p levels in RCC tissues were detected by MS-PCR analysis.12 *μ*l of PCR product was run on 2.2% agarose gel, stained with GelRed, and visualized under Tanon UV illumination. The number (1, 2, 3) indicated pairs of patients’ samples, with each pair comprising of one tumor tissue (T) and one normal tissue (N). (**P<0.01, ***P<0.001). (**c**) qRT-PCR was used to detect the miR-106a-5p expression levels after treatment of 5-AzaDc for 5 days in 786-O (upper) and ACHN (lower) cell lines. The results were all carried out in triplicate.

On the grounds of the correlations between hypermethylation and tumor-suppressor miRNAs in cancers,^25^ we next investigated whether the miR-106a-5p gene promoter region was hypermethylated in RCC. Methylation-specific PCR (MS-PCR) analysis was performed on 786-O and ACHN cell lines in the presence or absence of 5-aza-2′-deoxycytidine (5-AzaDc, DNA demethylating agent) for 5 days. The results showed that the methylated levels significantly declined along with the unmethylated levels increased under 5-AzaDc treatment (Figure 6a), validating the regulation of miR-106a-5p gene promoter by methylation *in vitro*. To further discuss the methylation condition of miR-106a-5p gene promoter region *in vivo*, three pairs of tissue samples (RCC tissues and normal adjacent tissues) from 30 pairs of RCC patients were randomly allotted for MS-PCR and the results revealed higher methylation and lower unmethylation levels in the 3 cases of tumor tissues *versus* normal adjacent tissues (Figure 6b). Yi, *et al.*^26^ reported that the downregulation of miRNA in tumor cell lines might be attributed to the high methylation level of its gene promoter region. To figure out the changes of miR-106a-5p expression levels under the condition of demethylation by 5-AzaDc for 5 days, qRT-PCR was performed and the results showed that miR-106a-5p levels were remarkably upregulated in both 786-O and ACHN cell lines (Figure 6c). It suggested that the hypermethylated gene promoter region of miR-106a-5p in RCC might be partially responsible for its downregulated expression.

## Discussion

It has been reported that miR-106a-5p is downregulated in osteosarcoma, colon cancer and astrocytoma and acts as a tumor suppressor,^17, 18, 19^ whereas its expression level and role in RCC remain unveiled. In this study, we found that miR-106a-5p was downregulated in both RCC tissues and cell lines. MiR-106a-5p belongs to miR-17 family, members of which has been reported to facilitate cancer development by promoting cell proliferation, inhibiting apoptosis and inducing tumor angiogenesis.^27, 28^ Besides, researchers have found that other members of miR-17 family are able to promote tumor invasiveness and regulate cell metastasis.^29, 30^ In this study, our results from transwell assays showed that miR-106a-5p could significantly attenuate RCC cell migration and invasion and wound healing abilities. Moreover, miR-106a-5p levels were found negatively correlated with TNM staging in RCC patients, with miR-106a-5p levels further downregulated in advanced TNM stages (III/IV) than in early stages (I/II). We conclude that miR-106a-5p plays a pivotal role in tumorigenesis and is related with RCC staging.

PAK5 is a serine/threonine kinase downstream of Rho GTPases.^31^ Many studies found that PAK5 possessed a crucial role in promoting cell migration and invasion in tumorigenesis,^21, 22, 23^ which preluded our exploration of whether miR-106a-5p could affect the migration and invasion of RCC cells via PAK5. PAK5 contains a highly conserved p21-GTPase-binding domain, which facilitates the interaction with GTP-binding Cdc42 (a Rho GTPase), and its cooperation with other Rho GTPases-like Rho and Rac may result in regulated cytoskeleton, thus altering the cell dynamics and morphology.^32, 33, 34^ In addition, PAK5-mediated signal pathways such as PAK5-Egr1-MMP2 are also involved in tumor cells migration and invasion.^24, 35^ In view of our findings upon inverse correlation between miR-106a-5p and PAK5, we addressed the role of PAK5 in miR-106a-5p-mediated regulation of RCC cells migration and invasion via transwell assays. As speculated, our results validated that miR-106a-5p regulated the migration and invasion of RCC cell lines by targeting PAK5.

It was validated that epithelial-mesenchymal transition (EMT) promoted cell invasion that led to tumor cell dissemination, and miR-106a-5p was discovered to exert a suppressive effect on the migration of osteosarcoma and astrocytoma cells,^17, 18, 36, 37^ we hypothesized that miR-106a-5p might be involved in EMT process (Supplementary Figure 2). The expression levels of two EMT markers were analyzed by western blot and the results showed that overexpressed miR-106a-5p (mimics) upregulated the N-cadherin expression and downregulated E-cadherin while the trends were reversed by the inhibition of miR-106a-5p (inhibitor), and our team are studying deep into it (Supplementary Figure 3).

Our *in vivo* studies showed that overexpressed miR-106a-5p in RCC cells inhibited the nodular metastasis in the lungs of nude mice *versus* negative control groups, which was in line with our *in vitro* experiments. Intriguingly in this study, we found that miR-106a-5p could not be able to exert significant effects on cell proliferation though its target PAK5 was reported to be involved in regulation of tumor cell proliferation.^38, 39^ It suggested complicated redundancies might exist downstream of miR-106a-5p but upstream of PAK5 which possessed a mystical power to recover potential regulation on RCC cell proliferation by PAK5.

Studies on epigenetic regulation indicated that in the tumor progression, even in early stages, the promoters of DNA loci encoding tumor suppressor miRNAs invariably harbor hypermethylated CpG islands.^26, 40^ Interestingly, we noted that the promoter region of miR-106a-5p gene was notably hypermethylated in RCC tissues in contrast to normal adjacent tissues, or rather, the downregulation of miR-106a-5p in RCC was partly attributed to methylation. The miRNAs biogenesis commences with processing of primary miRNAs (pri-miRNAs), in which RNA binding protein DGCR8 and the ribonuclease type III DROSHA are intricately involved. Pri-miRNAs can be methylated by methyltransferase-like 3 (METTL3) and render more recognizable by DGCR8, with miRNA maturation enhanced in a non-cell-type specific manner.^41, 42, 43, 44, 45, 46^ Therefore, we postulate that miR-106a-5p may be regulated by certain methyltransferase like METTL3, whereas further study is still requisite.

Taken together, our findings validated that miR-106a-5p was markedly correlated with RCC progression, in which might serve as a candidate biomarker. Our results both *in vivo* and *in vitro* demonstrated that miR-106a-5p could be a novel therapeutic target in the suppression of RCC metastasis, at least partially via the mediation of PAK5, with more profound mechanism awaiting further investigation.

## Materials and methods

### Cell culture and clinical samples

The human RCC cell lines OSRC-2, 786-O, ACHN, Ketr-3 and normal human renal tubular epithelial cell line HK2 were obtained from the Cell Bank, China Academy of Sciences (Shanghai, China). OSRC-2 and 786-O cells were maintained in RPMI 1640 medium (Hyclone, Logan, USA) supplemented with 10% (v/v) fetal bovine serum (FBS; Gibco, Shanghai, China). ACHN, Ketr-3 and HK2 cells were cultured in Dulbecco’s modified Eagle’s medium (Hyclone) supplemented with 10% (v/v) FBS. All cells were cultivated at 37 °C with 5% CO_2_.

All the tissue samples were obtained from the Affiliated Hospital of Xuzhou Medical University with approval of the Review Board of the Affiliated Hospital of Xuzhou Medical University. All investigations involving humans were performed in accordance with the World Medical Association Declaration of Helsinki. All the details of samples used in this experiment were listed in Supplementary Table 1.

### Transfections and stable cell line generation

786-O and ACHN cells were transiently transfected with 20 nM miR-106a-5p mimics, mimics negative control (mimics-nc), miR-106a-5p inhibitor, inhibitor negative control (inhibitor-nc) and small interfering RNA of PAK5 (si-PAK5, Genepharma, Shanghai, China) using siLentFect Lipid Reagent (Bio-Rad, Hercules, CA, USA) according to the manufacturer’s instructions except that pcDNA3.1-PAK5 plasmids (overexpressed PAK5) were transfected by Invitrogen Lipofectamine 2000 Reagent (Thermo Fisher, Shanghai, China). Following transfection of 24 to 48 h, cells were collected for subsequent investigation. The miR-106a-5p mimics-nc-786-O cells (Ctrl-786-O cells) and miR-106a-5p mimics-786-O cells (mimics 786-O cells) were established by infected with lentiviruses, in which miR-106a-5p mimics-nc expression vectors and miR-106a-5p mimics expression vectors were respectively packed (GenePharma). Target cells were transfected with lentivirus for 48 h followed by selection with puromycin (Vicmed) for 30 days. The sequences of all oligoes used for transfections were as follows: of mimics-nc, sense, 5′-UUCUCCGAACGUGUCACGUTT-3′, antisense, 5′-ACGUGACACGUUCGGAGAATT-3′, of mimics, sense, 5′-AAAAGUGCUUACAGUGCAGGUAG-3′, antisense, 5′-ACCUGCACUGUAAGCACUUUUUU-3′, of inhibitor-nc, 5′-CAGUACUUUUGUGUAGUACAA-3′, of inhibitor, 5′-CUACCUGCACUGUAAGCACUUUU-3′ (GenePharma).

### RNA isolation, reverse transcription and quantitative real-time PCR (qRT-PCR)

Total RNA was extracted with Trizol Reagent (Thermo Fisher) and reverse transcription of miRNA and mRNA performed with the SYBR PrimeScript miRNA RT-PCR Kit and PrimeScript RT Master Mix (Takara, Dalian, China) according to the manufacturers’ instructions. MiRNA levels were determined by SYBR Green II (Takara) with a 7500 Real-time PCR System (Life Technologies, NY, USA). Relative quantitation of miR-106a-5p was normalized to U6 levels. The primer sequences were as follows: miR-106a-5p forward, 5′-GATGCTCAAAAAGTGCTTACAGTGCA-3′ miR-106a-5p reverse, 5′-TATGGTTGTTCTGCTCTCTGTCTC-3′ (GenePharma); U6 forward, 5′-GCTTCGGCAGCACATATACTAAAAT-3′ U6 reverse, 5′-CGCTTCACGAATT TGCGTGTCAT-3′ (Sangon, Shanghai, China). All reactions were run in triplicate, and the relative gene expression was calculated using the comparative threshold cycle (Ct) method (relative gene expression=2^-(ΔCtsample−ΔCtcontrol)^).

### Cell migration and invasion assays (Transwell assay)

Modified two-chamber plates with a pore size of 8 *μ*m were employed for cell migration and invasion assessments. The transwell inserts (Corning Incorporated, New York, USA) without or with Matrigel (BD Biosciences, San Jose, CA, USA) coating were applied for migration or invasion assays, respectively. Cells after transfection were planted into the transwell inserts. After modest cultivation, cells were immobilized by 4% of paraformaldehyde solution (Vicmed) followed by crystal violet (Vicmed) staining. Then, we used a Nikon digital camera to take pictures with the magnification of × 100 followed by ImageJ software to compute the numbers of cells penetrating through the pores. All experiments were performed three times in triplicate.

### Cell proliferation assay (CCK-8 assay)

Cell proliferation was gauged with the Cell Counting Kit-8 (CCK-8) kit (Vicmed). 24 h after transfection, cells were plated in a 96-well microplate (Corning) in triplicate and incubated at 37 °C with 5% CO_2_. 10 *μ*l CCK-8 solution with 100 *μ*l serum-free medium was added to each well at 24 h, 48 h and 72 h, respectively, followed by incubation for 2 h. The absorbance at 450 nm was measured to calculate the cell viability by a multi-function enzyme-linked analyzer (Biotek Instruments, Winooski, VT, USA). Experiments were repeated three times.

### EdU staining

The EdU staining was performed following keyFlour488 Click-iT EdU imaging detection kit (KeyGEN Biotech, Nanjing, China) instrument. 786-O and ACHN cells were transfected with mimics or inhibitor and seeded on coverslips in 24-well plates. Then cells were immobilized by 3.7% neutral methanol for 15 min and permeabilized with 0.1% Triton X-100 for 15 min. Next, cells were incubated with 10 *μ*M EdU for 30 min. After treating with 3% BSA in PBS, Hoechst33342 was used for nuclear staining (Blue). Cell numbers of EdU-staining were counted per field (Green). Data are shown from a typical experiment performed in triplicate.

### Dual luciferase reporter assay

The PAK5-WT 3′-UTR firefly luciferase construct (psiCHECK2-PAK5-WT 3′-UTR) was generated by insertion of a 264 bp fragment of human PAK5 3′-UTR into the Xho I/Not I sites of the psiCHECK2 Luciferase Report vector. The psiCHECK2-PAK5 mut 3′-UTR construct was generated by mutation of the complementary seed sequence to the miR-106a-5p binding region of PAK5 (Ibsbio, Shanghai, China). 786-O and ACHN cells were co-transfected psiCHECK2-PAK5 WT 3′-UTR or psiCHECK2-PAK5 mut 3′-UTR luciferase reporter plasmids with miR-106a-5p mimics-nc or mimics using Invitrogen Lipofectamine 2000 Reagent, respectively. The activities of firefly or renilla fluorescence were assessed by Dual-Luciferase Reporter Assay System at the end of 48 h transfection (Promega, Beijing, China). Each treatment was performed in triplicate in three independent experiments.

### Wound healing assays

786-O and ACHN cells in complete media (supplemented with 10% FBS) that were grown to confluence in 6-well plates (Corning) were wounded by scratching the cell monolayer with a disposable 200 *μ*l pipette tip. A Nikon digital camera with magnification of × 100 screened all the pictures of wound healing assays. The wounds pictures then were photographed per 3 h and closures were recorded regularly. In Figure 2e, we chose the pictures of 12 h time point for their marked comparisons. Experiments were performed in thrice.

### Western blot analysis

Total protein was distilled using RIPA lysis buffer (Beyotime, Shanghai, China) and thereby gauged by Enhanced BCA Protein Assay Kit (Beyotime). Equivalent proteins from each sample were separated by SDS-PAGE electrophoresis and transferred to nitrocellulose blotting membranes (Pall Corporation, Mexico). Subsequently, the membranes were blocked with tris-buffered saline containing Tween-20 (TBST, 150 mM NaCl, 20 mM Tris-HCl pH 8.0, 0.05% Tween-20) and 5% non-fat dry milk for 2 h at room temperature (r/t) and incubated with specific primary antibodies: anti-PAK5 (1: 500, Abcam, Shanghai, China, catalog: ab110069) or anti-*β*-actin (1: 1000, ZSGB-BIO, Beijing, China, catalog: TA-09) for overnight at 4 °C. Having been rinsed thrice, the membranes were incubated with horseradish peroxidase (HRP)-conjugated secondary antibodies (1:10000, Vicmed, catalog: VA001 and VA002) for 2 h at r/t. Protein bands were determined using a Tanon High-sig ECL Western Blotting Substrate (Tanon, Shanghai, China) and analyzed by Image analysis software (Tanon). The grey levels of western blot analysis were measured and quantified by ImageJ software. The grey levels of PAK5 were normalized to those of *β*-actin and expressed as a percentage of control. All experiments were performed in triplicate.

### Tail vein metastasis assay

The BALB/c female nude mice were customized by HFK Bioscience (Beijing, China) and randomized into two groups (*n*=7 for each). 3 × 10^6^ Ctrl-786-O cells and mimics 786-O cells suspended in 150 *μ*l PBS were intravenously inoculated into the tail veins. 2 months thereafter, mice in both groups were sacrificed, with the lungs isolated for photography and immersed in RNA sample conservation medium (Vicmed) at 4 °C. Afterwards, extractions of the RNA and proteins of the resected lungs were performed using Trizol Reagent (Thermo Fisher) for subsequent experiments. All animal experiments were in conformance with the ARRIVE (Animal Research: Reporting of *In Vivo* Experiments) guidelines and in accordance with the National Institutes of Health Guide for the Care and Use of Laboratory Animals.

### 5-aza-2’-deoxycytidine treatment

786-O and ACHN cells were seeded in 10 cm petri dishes and exposed to 5 *μ*M of 5-aza-2’-deoxycytidine (5-AzaDc, Sigma-Aldrich, MO, USA) at 60% confluence. Afterwards, the dishes of cells were incubated for 5 days in complete medium with 5-AzaDc, which were replaced by fresh culture medium with 5-AzaDc at an interval of 24 h.

### Methylation-specific PCR (MS-PCR)

Genomic DNA from tissue specimens and cell lines were extracted with TIANamp Genomic DNA Kit (Tiangen Biotech, Beijing, China) and converted by bisulfite with EpiTect Bisulfite Kit (Qiagen, Duesseldorf, Germany) in compliance with the prior protocol.^47^ MS-PCR was performed using a methylation-specific kit (Tiangen Biotech) on purified DNA with methylated primers and unmethylated primers as follows: 95 °C for 5 min, followed by 35 cycles at 94 °C for 20 s, 60 °C for 30 s, 72 °C for 20 s, and a final extension at 72 °C for 5 min. The resultant products were visualized by agarose gel electrophoresis with GelRed (Vicmed). All experiments were performed in triplicate with the details of methylated and unmethylated primers sequences were as follows: methylated primer, forward: 5′-TATTAGGTAAGGGTGTGAGAGAC-3′, reverse: 5′-ACTAAACAATCACTCTCCAATCC-3′, unmethylated primer, forward: 5′-GTATTAGGTAAGGGTGTGAGAGAT-3′, reverse: 5′-ACTAAACAATCACTCTCCAATCC-3′.

### Statistical analysis

Statistical analysis was performed by SPSS v.16.0 software (Shanghai, China) and images were acquired with GraphPad Prism 5 Software (La Jolla, CA, USA). Data are represented as the mean±standard deviation (SD). The between-group differences were evaluated using Student’s *T* test or one-way ANOVA, and the correlation analyses were using Pearson’s correlation analyses, with *P*<0.05 defined as statistically significant (*P<0.05, **P<0.01, ***P<0.001).

## Publisher’s Note

Springer Nature remains neutral with regard to jurisdictional claims in published maps and institutional affiliations.
