# Supplementary material for: MiR-106a-5p inhibits the cell migration and invasion of renal cell carcinoma through targeting PAK5
Source: Cell Death Dis. 2017 Oct 26;8(10):e3155–. doi: 10.1038/cddis.2017.561 (PMC5680926; doi:10.1038/cddis.2017.561)
Supplement: Supplementary Figure 1 [file cddis2017561x1.pdf]

Supplementary Figure 1

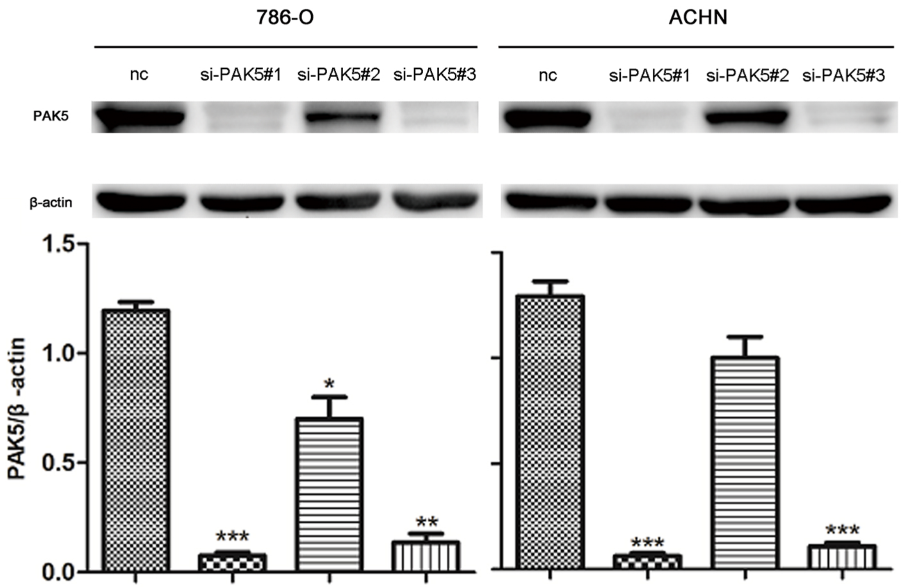

Supplementary Figure 1: The effectiveness of small interfering RNA of PAK5. Western blot analysis was used to detect the PAK5 protein level in 786-O (left) and ACHN (right) cells after transfection of three small interfeing RNA of PAK5. β-actin was used as an internal control. (*$P < 0.05$, **$P < 0.01$, ***$P < 0.001$)
