# Supplementary material for: MiR-106a-5p inhibits the cell migration and invasion of renal cell carcinoma through targeting PAK5
Source: Cell Death Dis. 2017 Oct 26;8(10):e3155–. doi: 10.1038/cddis.2017.561 (PMC5680926; doi:10.1038/cddis.2017.561)
Supplement: Supplementary Figure 2 [file cddis2017561x2.pdf]

**Supplementary Figure 2**

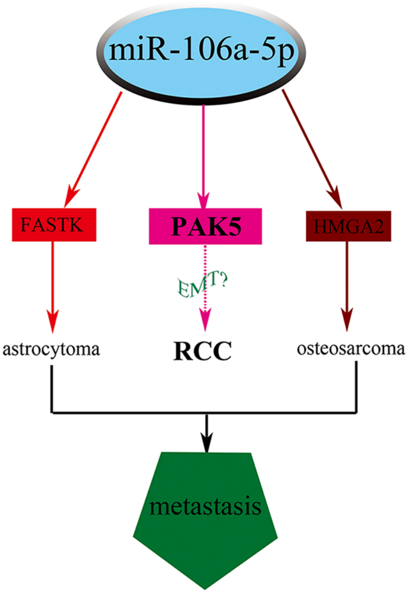


**Supplementary Figure 2: The relationships between miR-106a-5p and metastasis.** FASTK and HMGA2 are direct targets found in astrocytoma and osteosarcoma respectively, and in this study we found PAK5 was directly targeted by miR-106a-5p.
