# Supplementary material for: MiR-106a-5p inhibits the cell migration and invasion of renal cell carcinoma through targeting PAK5
Source: Cell Death Dis. 2017 Oct 26;8(10):e3155–. doi: 10.1038/cddis.2017.561 (PMC5680926; doi:10.1038/cddis.2017.561)
Supplement: Supplementary Figure 3 [file cddis2017561x3.pdf]

## Supplementary Figure 3

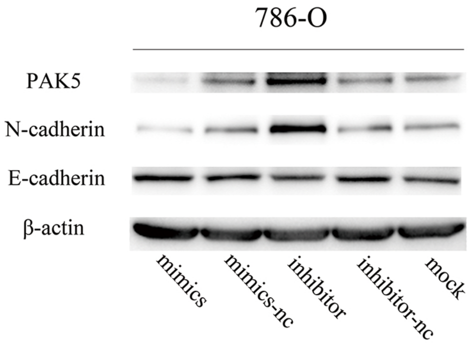


**Supplementary Figure 3: The possible interactions of miR-106a-5p with EMT markers.** After transfection with mimics, the protein levels of PAK5 and N-cadherin were downregulated accompanying E-cadherin was upregulated. On the contrary, inhibition of miR-106a-5p could upregulate the protein levels of PAK5 and N-cadherin following with downregulation of E-cadherin. The mock group was used as a control.
